# Severe nonbacterial preseptal cellulitis from adenovirus detected via pooled meta‐genomic testing

**DOI:** 10.1002/ccr3.3468

**Published:** 2020-11-03

**Authors:** Saurabh Bansal, Divya P. Nimmatoori, Namrata Singhania, Rone Chun Lin, Chandra Mouli Nukala, Anil K. Singh, Girish Singhania

**Affiliations:** ^1^ Department of Internal Medicine University of Illinois College of Medicine at Peoria Peoria IL USA; ^2^ Department of Internal Medicine GreenField Health Portland OR USA; ^3^ Department of Hospital Medicine Mount Carmel East Hospital Columbus OH USA; ^4^ Department of Infectious Disease University of Illinois at Peoria Peoria IL USA; ^5^ Department of Hospital Medicine CHI St Vincent Infirmary Little Rock AR USA; ^6^ Department of Internal Medicine Geisinger Community Medical Center Scranton PA USA; ^7^ Division of Nephrology and Hypertension University of Utah Salt Lake City UT USA

**Keywords:** adenovirus, hemorrhagic conjunctivitis, microbial cell‐free DNA, preseptal cellulitis, steroids

## Abstract

Preseptal cellulitis is a serious diagnosis that can progress to postseptal cellulitis leading to grave consequences. Clinically, viral and bacterial cellulitis can be indistinguishable from each other. Using rapid DNA/RNA sequencing can be helpful.

## Graphical abstract

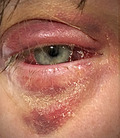

## 1 INTRODUCTION

Acute epibulbar infections are one of the most frequently diagnosed ophthalmologic infections. They are usually self‐limiting but can lead to a viral preseptal cellulitis mimicking a severe bacterial infection. Early diagnosis is important to shorten the course of recovery.

Preseptal cellulitis is a soft‐tissue infection that develops secondary to trauma, coryza, or local skin inflammation. Infections are usually bacterial with hemophilus influenzae, staphylococci, and streptococci being the most common pathogens.^1^, ^2^ Although viruses are rarely implicated in the etiology, varicella is known to cause preseptal cellulitis via an eyelid infection without prior respiratory involvement. Rarely, adenovirus eye infections are complicated by bulbar cellulitis. Herein, we present a case of a young man with an adenovirus‐associated preseptal cellulitis diagnosed by pooled meta‐genomic testing and successfully treated with topical steroids.

## 2 CASE DESCRIPTION

**FIGURE 1:**
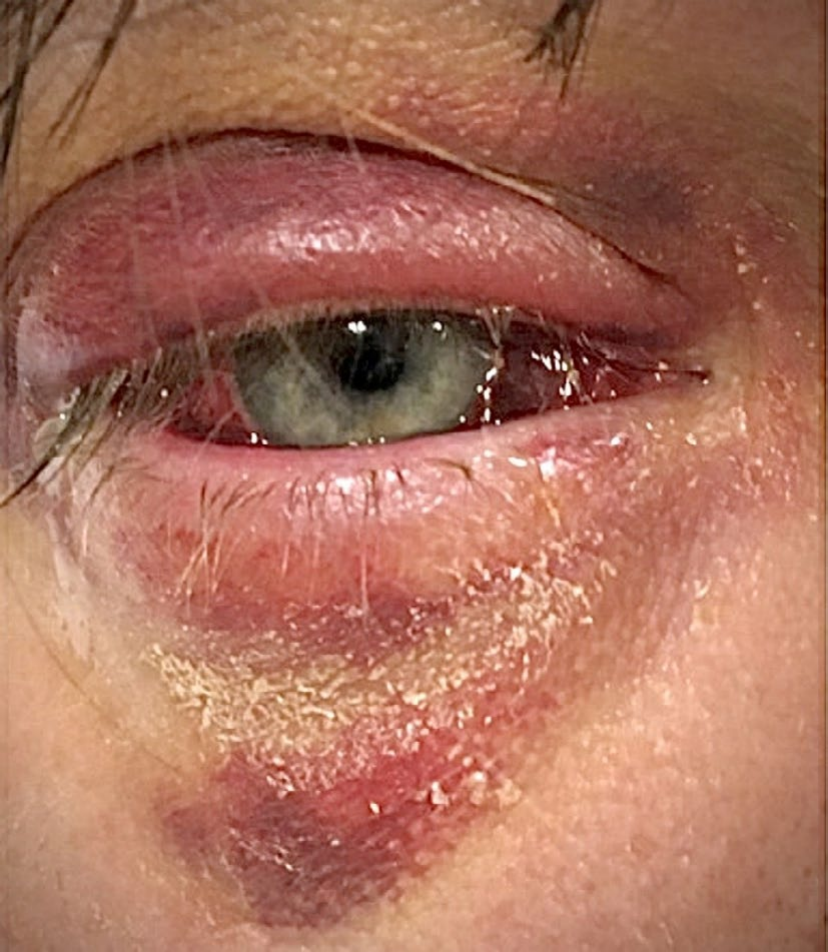
Upper and lower right eyelid showing redness, ecchymoses, swelling, crusting, and discharge from the corner of his right eyelid along with diffuse subconjunctival hemorrhage

A 36‐year‐old man with history of asthma, hypertension, uncontrolled diabetes, and obesity presented with sudden onset of redness and swelling in the right eye that started 6 days prior to hospitalization and was gradually getting worse. He initially went to an urgent care where he was prescribed cephalexin 500 mg twice a day. After 2 days, he did not notice significant relief and developed a new blurry vision associated with photophobia in the right eye. Later, he spiked a fever of 103°F at which point he decided to come to the hospital and got admitted. He denied reports of trauma, local injury, swimming pool exposure, and use of contact lens. He denied any recent sick contacts and lives with his spouse and three young kids, none of whom were sick in recent times. He reported a monogamous relationship with his wife and denied any history of sexually transmitted diseases. He denied intravenous drug use, smoking, or alcohol use. He had two pet dogs, none of whom were sick with similar illness. His vital signs were stable at presentation except for fever as mentioned above. On examination, he had severe redness of palpebral conjunctiva with diffuse subconjunctival hemorrhage. Upper and lower right eyelid showed redness, ecchymoses, swelling, crusting, and discharge from the corner of his right eyelid (Figure 1).

His left eye was normal. Pupils were equally reactive to light on both sides. Intraocular pressure measured by tono‐pen was within normal limits. Visual field testing showed mild generalized constriction of visual field of the right eye. Bilateral corneas, anterior chamber, iris, and lenses were grossly normal without any abnormality. Fundoscopic examination via indirect ophthalmoscopy after pupillary dilatation showed normal vitreous and absence of retinal or optic disc changes. All cranial nerves were intact, and extraocular muscles showed full range of motion with tenderness on full extension. Motor, sensory examination, and reflexes were equal and bilaterally symmetrical.

**FIGURE 2:**
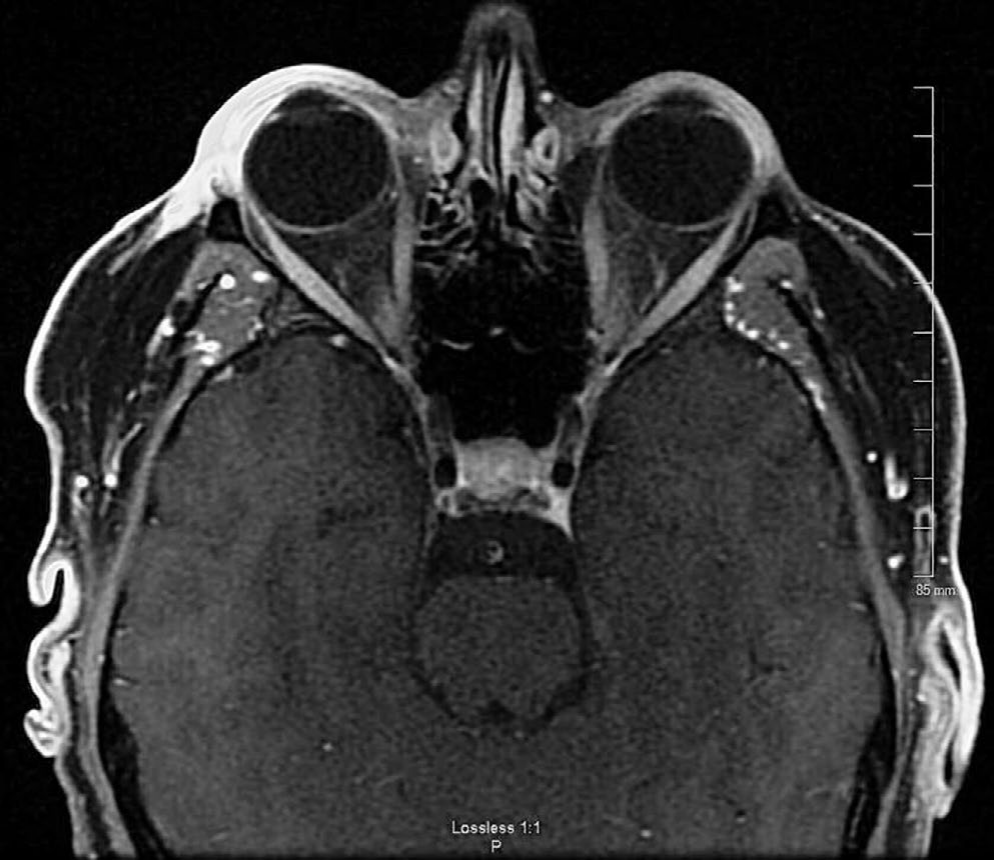
Magnetic resonance imaging (MRI) of the brain with contrast. Right eye showing significant subcutaneous tissue edema and enhancement. Postseptal area, eyeball, and optic nerve appear within normal limits

Upon admission, he was started on broad‐spectrum antibiotics. White blood cell count, hemoglobin, platelets, serum electrolytes, serum creatinine, and liver function tests were all within normal limits except for an elevated blood sugar of 268 mg/dL (normal range [NR]: 70‐110 mg/dL). Procalcitonin was normal at 0.04 ng/mL (NR: <.1 ng/mL), erythrocyte sedimentation rate was elevated at 54 mm/hour (NR: 1‐13 mm/hour), and C‐reactive protein was 0.94 mg/dL (NR: <1 mg/dL). HIV 1&2 antigen and antibody test were negative. Magnetic resonance imaging (MRI) of face showed preseptal soft tissue thickening consistent with cellulitis. Ocular globe, postseptal space, ocular lenses, extraocular muscles, optic nerve sheath, lacrimal glands, and retro‐bulbar fat appeared normal with no involvement from infection (Figure 2).

Blood cultures returned negative after 5 days of incubation. Serum sample was sent for microbial cell‐free DNA test also known as meta‐genomic testing. It showed 459 DNA molecules per microliter (MPM) of human adenovirus D in the serum sample with normal range of <10 MPM. The patient was started on topical steroids, and he responded well with significant improvement in signs and symptoms within first 3 days. Antibiotics were discontinued, and the patient was discharged home with no sequelae at the follow‐up visit.

## 3 DISCUSSION

The adenoviruses are a family of a globally distributed, perennial, DNA viruses that commonly cause a self‐limiting febrile illnesses in children. It is the most common cause of viral conjunctivitis in children older than 6 years.^3^ Fatal infections can occur in immunocompromised hosts with a low cell‐mediated immunity and are rarely seen in healthy individuals.^4^ Over 60 human adenovirus serotypes, further classified into seven species (A‐G) based on biologic properties, have been described. Species B serotypes 3 and 7 are the most common. Species B, C with serotypes 5, 7, 14, and 21 are primarily responsible for a severe infection.^5^ Infections are prevalent in daycare centers and crowded settings. The mode of transmission is via aerosol, fecal‐oral route, or contaminated fomites. Long periods of survival on inanimate surfaces and resistance to lipid disinfectants due to its nonenveloped structure contribute to a faster and wider spread. Most individuals have a serologic evidence of prior infection during the first decade of their life.^6^

The age and immunocompetency of the host dictate the range of clinical manifestations in the patient. While respiratory tract infections like pharyngitis, coryza, and pneumonia are common, gastrointestinal, ophthalmologic, and genitourinary involvement can also be seen. Adenovirus can cause severe hemorrhagic conjunctivitis which can mimic bacterial cellulitis leading to an unnecessary antimicrobial treatment such as seen with our patient.^7^ The illness usually lasts 5 days to 2 weeks with further lengthening in the case of a bacterial super‐infection. The classic presentation is that of a pharyngo‐conjunctival fever with follicular conjunctivitis, febrile pharyngitis, cervical adenitis, and associated systemic manifestation, leading to a clinically indistinguishable picture from the group A streptococcal infection.^8^ A whitish membrane seen on the palpebral conjunctiva (pseudomembrane) along with eyelid edema is a common physical examination sign seen in such patients.^7^ Ophthalmologic involvement in the form of epidemic keratoconjunctivitis, caused by species D serotypes 8, 19, and 37, is a serious infection characterized by blurry vision, bilateral conjunctivitis, preauricular adenopathy, and painful corneal opacities.^8^ The adenoviral disease spectrum may include an exanthem, hemorrhagic cystitis (species B serotypes 11 and 21), otitis media, pertussis‐like syndrome, bronchiolitis, myocarditis, viral myositis, acute respiratory distress syndrome, meningoencephalitis, hepatitis, tubulointerstitial nephritis, urethritis, neutropenia, and disseminated intravascular coagulation.^9^ Immunocompromised hosts, such as patients with hematopoietic stem cell or solid organ transplant, may have a higher risk of disseminated infection causing pneumonia, hepatitis, colitis, nephritis, and encephalitis.^4^

Adenoviral antigen or polymerase chain reaction assays can be used to detect the virus from an eye swab sample.^4^ Serum DNA testing via microbial cell‐free DNA test offers rapid identification of virus, especially when the viral vs bacterial diagnosis is in doubt, thus avoiding cumbersome viral culture which is not high yielding.^10^, ^11^ Next‐generation sequencing creates full sequences from the free or fragmented DNA present in the plasma and compares it with stored DNA sequences in the database to find an exact match. Such technique can scan for the presence of DNA of multiple organisms, including bacterial, fungal, parasitic, and viral in one serum sample.^12^ Shorter turnaround time when compared to traditional culture provides an additional benefit. The test has a diagnostic sensitivity of 92.9% and can detect a potentially sepsis causing organism three times more often than initial blood cultures. Currently, the sensitivity in detecting viral infections of distant organs via next‐generation sequencing is still unclear. Sensitivity is better for disseminated infections, where micro‐organism is expected to cross plasma. Early and accurate identification of viral etiology helps to de‐escalate antibiotics sooner thus reducing the adverse effects. It also allows initiation of topical steroids, which otherwise would be contraindicated in a typical bacterial infection, leading to faster recovery. While supportive therapy and steroids are mainstay in treatment, the re‐introduction of adenovirus vaccination in military recruits showed significant reduction in the incidence of adenovirus‐related febrile respiratory illness.^9^, ^13^

## 4 CONCLUSIONS

Preseptal cellulitis is a severe infection of the eye which is commonly caused by bacterial organisms but can rarely be viral. Physician should be aware of the various etiologies, as early detection of causative organism can lead to focused treatment with de‐escalation of antibiotics, along with appropriate use of topical steroids in cases of viral etiology. A pooled meta‐genomic testing with detection of microbial cell‐free DNA in plasma can be a useful test in identifying the causative organism early in the disease process.

## CONFLICT OF INTEREST

The authors do not have any conflict of interest to disclose.

## AUTHOR CONTRIBUTIONS

SB, DPN, and NS: contributed equally in preparing the manuscript and reviewed the literature. RCL, CMN and GS: critically revised the manuscript.

## ETHICAL APPROVAL

Ethics committee was not consulted for approval as it is a case report and all possible efforts were made to maintain complete anonymity.
